# Idiopathic Infantile Arterial Calcification: A Rare Cause of Sudden Unexpected Death in Childhood

**DOI:** 10.4061/2010/185314

**Published:** 2010-07-27

**Authors:** Susana Guimarães, José Manuel Lopes, José Bessa Oliveira, Agostinho Santos

**Affiliations:** ^1^Institute of Molecular Pathology and Immunology, University of Porto (IPATIMUP), Porto, Portugal; ^2^Department of Pathology, Hospital de São João, Porto, Portugal; ^3^Medical Faculty, University of Porto, Porto, Portugal; ^4^Legal Medicine Institute, North Delegation, Porto, Portugal; ^5^Abel Salazar Institute of Medical Sciences (ICBAS), University of Porto, Porto, Portugal

## Abstract

Unexpected child death investigation is a difficult area of forensic practice in view of the wide range of possible genetic, congenital, and acquired natural and nonnatural causes. Idiopathic infantile arterial calcification (IIAC) is a rare autosomic recessive disease usually diagnosed postmortem. Inactivating mutations of the ENPP1 gene were described in 80% of the cases with IIAC. We report a case of a 5-year-old girl submitted to a forensic autopsy due to sudden death and possible medical negligence/parents child abuse. Major alterations found (intimal proliferation and deposition of calcium hydroxyapatite around the internal elastic lamina and media of arteries; acute myocardial infarct, stenotic and calcified coronary artery; perivascular and interstitial myocardial fibrosis; and subendocardial fibroelastosis) were diagnostic of IIAC. We reviewed IIAC cases published in the English literature and highlight the importance of adequate autopsy evaluation in cases of sudden child death.

## 1. Introduction

Idiopathic infantile arterial calcification (IIAC) is a rare autosomic recessive disease (OMIM 208000) usually diagnosed postmortem. The reported cases in the literature are about 200, with 85% of the patients diagnosed in infancy and dying before 6 months of age due to the difficulty of an early diagnosis and the lack of a curative treatment [1]. So far, there are only eleven long-term survivors (survival >6 months) reported in literature [1], having three of these diagnosed during childhood. The sudden death of a child may raise medical negligence and parents child abuse suspicions, inasmuch as these latter deaths are less common than those attributed to sudden infant death syndrome (SIDS).

We aim to report one case of IIAC and discuss the implications of this rare cause of sudden childhood death.

## 2. Case Report

A five-year-old female child, without any relevant personal past medical or familial history, presented with vomits and anorexia, without fever or diarrhoea, in the last 24 hours. She was diagnosed as having tonsillitis by the family doctor and was medicated accordingly. A few hours later she had a syncope episode associated with facial cyanosis and incontinence that lasted about 10 minutes, and was admitted to the Hospital.

On admission, she was conscious, hemodynamically stable, tachypneic, tachycardic, and with low fever and facial petechiae.

The electrocardiography showed sinusal tachycardia and frequent extrasystoles. Chest X-ray showed radiological features consistent with congestive heart failure. Routine analytic results were within normal range, namely, absence of hypoglycaemia. Cerebral CT scan did not disclose abnormalities. 

A few hours later, the child developed cardiorespiratory arrest without response to the intensive care resuscitation procedures. A forensic autopsy was requested.

## 3. Pathologic Findings

The external examination revealed no dysmorphic features, being the biometrical parameters (weight, height, and cephalic perimeter) within normal range (percentile 50). Internal examination showed bilateral hypertrophy of tonsils with purulent material over the crypts. The heart weighted 150 grams (>97.5th centile for age/gender, 85 grams) with left ventricular hypertrophy. The pericardium, pulmonary, and systemic venous drainage were normal. The left ventricle showed patchy areas of discoloration and coalescent haemorrhagic suffusions in the interventricular septum. The valvular apparatus was macroscopically normal. The coronary arteries showed a normal anatomical pattern but were firm on palpation. On sectioning, the major branches of the coronary arteries were patent with no significant macroscopically apparent luminal narrowing, but the anterior coronary artery was gritty on cut, consistent with calcification. Major arteries (aorta, pulmonary, carotid, and renal arteries) were unremarkable.

**Figure 1:**
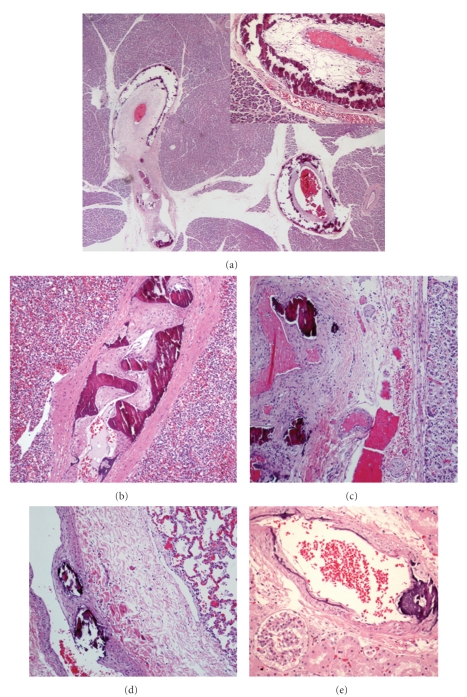
Calcification with severe stenosis of pancreatic ((a) H&E, x20, and inlay with intimal proliferation—x200), splenic ((b) H&E, x40), adrenal gland ((c) H&E, x40), lung ((d) H&E, x40), and renal ((e) H&E, x40) arteries.

**Figure 2:**
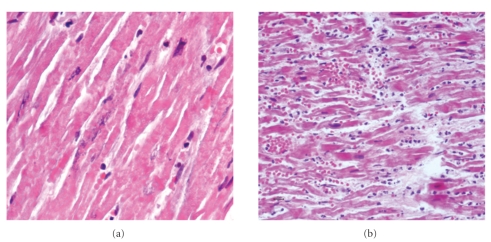
Acute heart infarction, with coagulative myocardial necrosis ((a) H&E, x400) and intersticial polymorphonuclear infiltrate ((b) H&E, x200).

**Figure 3:**
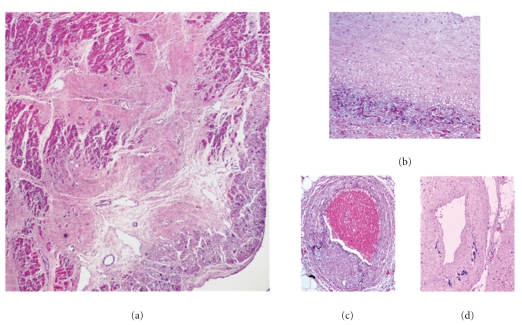
Myocardium interstitial fibrosis ((a) H&E, x100), subendocardial fibroelastosis ((b) H&E, x200), and coronary stenosis with wall calcification ((c, d) H&E, x200).

Histologically, there was calcification in the internal elastic lamina and media of medium-sized arteries in several organs, associated with intimal fibrous proliferation (Figure 1). Arterial walls lacked inflammatory infiltration. An acute myocardial infarction of the left ventricle septal wall, associated with stenosis and calcification of the anterior coronary artery is demonstrated (Figure 2). There were areas of perivascular and interstitial myocardial fibrosis, predominantly in the left ventricle, and subendocardial fibroelastosis, predominantly in the left auricle (Figure 3). Tonsillitis was confirmed. The toxicological study was negative.

## 4. Comment

One of the inclusion criteria for the Confidential Enquiry into Stillbirths and Deaths in Infancy (CESDI) for Sudden Unexpected Death in Infancy (SUDI) study of deaths is death occurring in the course of a sudden acute illness of less than 24-hour duration in a previously healthy infant, or a death that occurred after this if intensive care has been instituted within 24 hours of the onset of illness [7]. Cases of SUDI include congenital, inherited, and acquired diseases and cases in which the clinical history, death scene investigation, or autopsy examination raise concerns about poor parenting, neglect, physical abuse, and, in rare instances, homicide [7]. The older the child, the more complex are these issues. European groups as well as groups from USA and Australia proposed protocols for the management and investigation of cases of SUDI, which emphasize the multidisciplinary nature of these investigations and set the autopsy in the context of a greater whole [7]. Even though the developmental anatomy and physiology of infants and children are different, the sophisticated investigative methods developed during clinical, pathological, and laboratorial research into SIDS can and should be applied to sudden unexpected death in childhood (SUDC) [8].

Sudden death in childhood is rare. About 10% of paediatric deaths after 1 year of life are sudden and population-based studies put the individual age-related risk at around 1 : 20 000 to 1 : 50 000 per year. About half of these deaths are related to previously known abnormalities, with the most common being epilepsy, asthma, and cardiovascular diseases [9]. A third of the cases are diagnosed at necropsy, usually as infections or cardiac diseases. At least one out of six childhood sudden deaths remains unexplained [9].

The unexpected death in our case of a 5-year-old child, without any previous pathological condition, raised suspicion of medical negligence (incorrect diagnosis of tonsillitis and/or incorrect dosage of the medications prescribed) or child abuse in the form of parents' negligence, and therefore an autopsy was performed. Idiopathic infantile arterial calcification (IIAC) as a rare cause of infantile sudden death first described in 1901 [10]. In our case it occurred in a 5-year-old girl, due to acute infarction in a heart with chronic ischemic disease. IIAC displays calcification and intimal fibroblastic proliferation of medium- and large-sized arteries [1]. The most common cause of death is a rapidly progressive cardiac insufficiency [1]. The physical, radiological, and analytical examination can reveal a cardiac insufficiency and/or an acute infarction without other associated abnormalities, and may be suggestive of the diagnosis in pre- and postnatal settings [1].

Foetal ultrasonography is the modality of choice for the prenatal diagnosis of IIAC, usually showing hyperechogenicity of the walls of major arteries and signs of fetal hydrops with heart chamber hypertrophy and cardiomegaly [11], and this is recommended in future pregnancies of the affected families.

The most frequent autopsy findings are myocardial hypertrophy, subendocardial fibrosis, and firm and tortuous coronary arteries [1]. The major microscopical alterations, as in our case, are intimal proliferation and deposition of calcium hydroxyapatite around the internal elastic lamina and media of arteries [1, 12]. The intima hyperplasia with stenosis/occlusion of vascular lumina causes ischemia and organ dysfunction, with rapid and fatal evolution in the majority of reported cases [1]. 

It was postulated that the patients with arterial calcification without intimal proliferation might have spontaneous regression of the lesions or, at least, lack severe obstructive arteriopathy, and thus have longer survival and later disease manifestations. So far, there are eleven long-term survivors, as in our case, reported in literature (Table 1), most of them with an early diagnosis.

Inactivating mutations of the ENPP1 gene (ecto-nucleotide pyrophosphatase/phosphodiesterase 1) were described in 80% of the cases with IIAC. This gene is expressed on fibroblasts, chondrocytes, osteoblasts, and hepatocytes, having nucleotide pyrophosphohydrolase activity that generates inorganic pyrophosphate (PPi) [13]. PPi inhibits extracellular matrix calcification due to prevention of hydroxyapatite deposition. The reduction in the levels of PPi promotes calcification of the extracellular matrix, especially in the artery walls [13]. Some patients have late manifestations of the disease without mutations in the ENPP1 gene, probably due to alterations of other still unknown gene(s) [13]. In our case it was not possible to evaluate ENPP1 gene alterations. 

Early diagnosis and available treatment [1, 6] may help control arterial calcification and ischemic damage of patients. Nevertheless, IIAC remains a clinical, pathological and genetically heterogeneous disease, without any curative treatment.

## 5. Conclusion

Unexpected childhood death investigation is a difficult area of forensic practice in view of the wide range of possible genetic, congenital, and acquired natural and non-natural causes. Sudden death in less than 24 hours after a diagnosis of a common disease may raise concerns of parents' negligence or/and child abuse or rare diseases such as idiopathic infantile arterial calcification (IIAC). Therefore, an adequate autopsy examination is mandatory in all infant/child sudden deaths and both clinicians and pathologists must be aware of this entity when they are presented with a suggestive clinical background [14]. Because of the genetic aetiology, once the diagnosis is made, the family should be informed and genetic counselling offered.

## Figures and Tables

**Table 1:** Long-term survivors with idiopathic infantile arterial calcification.

Author	Age at diagnosis	Sex	Family history	Followup age/status	Cardiovascular findings	Treatment
Gleason et al. [2]	5 years	M	No	5 years/D	CHF	None
Gleason et al. [2]	2 months	M	Yes	5 years/A	NA	Steroid Thyroid estrogen
Gleason et al. [2]	Birth	F	Yes	3 years/A	None	Diphosphonates
Gleason et al. [2]	2 weeks	F	No	7 years/A	Pulmonary stenosis	None
Gleason et al. [2]	3 weeks	F	No	22 years/A	Angina; myocardial infarction	Digoxin Disopyramide
Gleason et al. [2]	10 days	M	No	2 years/A	CHF, HT	Diphosphonates Digoxin
Gleason et al. [2]	Birth	M	Yes	6 years/A	Arterial calcifications	Digoxin Diuretics
Rutsch et al. [3]	5 days	M	Yes	2,5 years/A	CHF	Antifailure Diphosphonates
Sebire and Sheppard [4]	11 years	F	No	11 years/D*	CHF	Dobutamine
Patel et al. [5]	2,5 years	F	No	4,5 years/A	Headaches; failure to thrive	Anti-failure
van der Sluis et al. [6]	Birth	M	Yes	25 years/A	None	Diphosphonates
Our case (2008)	5 years	F	No	5 years/D*	CHF	None

CHF: cardiac heart failure; HT: hypertension; A: Alive; D: Dead; NA: nonavailable; *Acute myocardial infarction.

## References

[1] Glatz AC, Pawel BR, Hsu DT, Weinberg P, Chrisant MRK (2006). Idiopathic infantile arterial calcification: two case reports, a review of the literature and a role for cardiac transplantation. *Pediatric Transplantation*.

[2] Gleason MM, Weber HS, Cyran SE, Baylen BG, Myers JL (1994). Idiopathic infantile arterial calcinosis: intermediate-term survival and cardiac sequelae. *American Heart Journal*.

[3] Rutsch F, Schauerte P, Kalhoff H, Petrarulo M, August C, Diekmann L (2000). Low levels of urinary inorganic pyrophosphate indicating systemic pyrophosphate deficiency in a boy with idiopathic infantile arterial calcification. *Acta Paediatrica*.

[4] Sebire NJ, Ramsay A, Sheppard M (2002). Idiopathic arterial calcification presenting with cardiac failure and sudden death in an 11-year-old girl. *Pediatric and Developmental Pathology*.

[5] Patel M, Andronikou S, Solomon R, Sinclair P, McCulloch M (2004). Idiopathic arterial calcification in childhood. *Pediatric Radiology*.

[6] Van Der Sluis IM, Boot AM, Vernooij M, Meradji M, Kroon AA (2006). Idiopathic infantile arterial calcification: clinical presentation, therapy and long-term follow-up. *European Journal of Pediatrics*.

[7] Howatson AG (2006). The autopsy for sudden unexpected death in infancy. *Current Diagnostic Pathology*.

[8] Krous HF, Chadwick AE, Crandall L, Nadeau-Manning JM (2005). Sudden unexpected death in childhood: a report of 50 cases. *Pediatric and Developmental Pathology*.

[9] Wren C (2002). Sudden death in children and adolescents. *Heart*.

[10] Bryant JH, White WA (1901). Case of calcification of the arteries and obliteration endarteritis associated with hydronephrosis in a child aged six months. *Guys.Hospital Reports*.

[11] Nasrallah FK, Baho H, Sallout A, Qurashi M (2009). Prenatal diagnosis of idiopathic infantile arterial calcification with hydrops fetalis. *Ultrasound in Obstetrics and Gynecology*.

[12] Cheng K-S, Chen M-R, Ruf N, Lin S-P, Rutsch F (2005). Generalized arterial calcification of infancy: different clinical courses in two affected siblings. *American Journal of Medical Genetics*.

[13] Numakura C, Yamada M, Ariyasu D (2006). Genetic and enzymatic analysis for two Japanese patients with idiopathic infantile arterial calcification. *Journal of Bone and Mineral Metabolism*.

[14] Hault K, Sebire NJ, Ho SY, Sheppard MN (2008). The difficulty in diagnosing idiopathic arterial calcification of infancy, its variation in presentation, and the importance of autopsy. *Cardiology in the Young*.

